# Complementary use of GCxGC–TOF–MS and statistics for differentiation of variety in biosolid samples

**DOI:** 10.1007/s00706-018-2221-z

**Published:** 2018-08-09

**Authors:** Hubert Byliński, Tomasz Dymerski, Jacek Gębicki, Jacek Namieśnik

**Affiliations:** 1 Department of Analytical Chemistry, Faculty of Chemistry, Gdańsk University of Technology, Gdańsk, Poland; 2 Department of Chemical and Process Engineering, Faculty of Chemistry, Gdańsk University of Technology, Gdańsk, Poland

**Keywords:** Gas chromatography, Mass spectroscopy, Odoriferous substances, Biosolid samples, Wastewater treatment plant

## Abstract

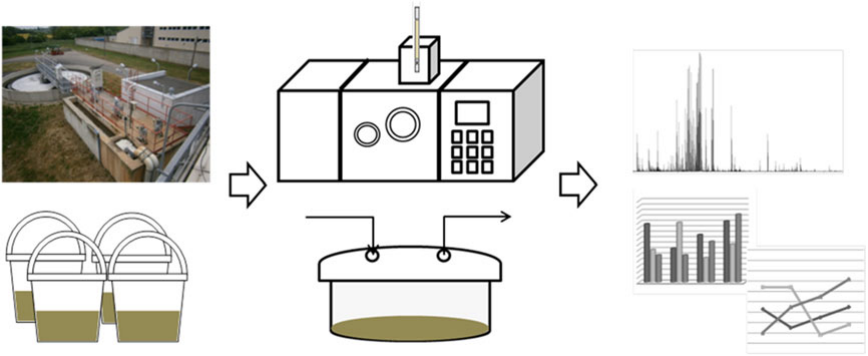

## Introduction

Operation of wastewater treatment plants is accompanied by generation of biosolid cakes, which are the sources of numerous hazardous chemical compounds, including malodorous ones [1–3]. The substances released during processing of the biosolids are complex mixtures of volatile organic compounds, with a significant contribution of organosulfur compounds, aldehydes, ketones, esters, and alcohols [4, 5]. Their content is strongly dependent on size of the city agglomeration and industrialization of the regions, from which sewage is collected as well as on a treatment technology employed. Selection of the optimum technology of biosolid cake processing should take into account changes in concentration level of particular chemical compounds. It is a very difficult task in case of the waste, including biosolids, generated in the wastewater treatment plants; the reason is variety in chemical composition of the material supplied [6].

The main unit operations, such as biosolid stabilization, composting, dewatering, drying, or thermal processing, are carried out in the wastewater treatment plants to limit negative environmental impact [7]. Execution of these processes is indispensable for further processing of the biosolid cakes; however, it does not ensure total removal of all potentially hazardous chemical and odoriferous compounds [8]. The investigations on the determination of the compounds released from the biosolid cakes show that their variety significantly hinders identification of those, which have the most negative influence on the environment. It is also difficult to point these compounds, which have the biggest impact on nuisance that affects the employees of the wastewater treatment plants and the residents of the areas neighbouring such municipal objects. That is why, it is necessary to elaborate a methodology of determination of the compounds released from the biosolid cakes, based on the instrumental tools available. This procedure should also utilize statistical and chemometric methods available. Significant optimization possibilities are created by statistical tests, which make it possible to define mutual correlation between investigated samples, including analysis of variance [9–12].

One of the technical solutions enabling measurement of emission of volatile odoriferous compounds, released from solid or liquid specimens surfaces (including the biosolid cakes), is application of the enclosure chambers [13]. In 1986, the Unites States of America Environmental Protection Agency (US EPA) issued a document regulating emission measurements using a flux hood chamber [14]. Its operation consists in: isolation of the investigated surface, blowing with high purity gas, and then measurement of the analytes using selected instrumental solution [15]. Chromatographic techniques, mainly gas chromatography coupled with mass spectrometry, are the most frequently used for determination of chemical composition of odoriferous gas mixtures. Popularity of this solution stems from the possibility of measurement of the components present at trace concentration level in the samples of various origin and composition [16, 17]. In the case of separation of complex mixtures of chemical compounds, one-dimensional chromatographic analysis can be insufficient for the identification of a wide array of the substances present in the samples. An alternative solution is two-dimensional gas chromatography coupled with time-of-flight mass spectrometry (GCxGC–TOF–MS), which is characterized by better resolution than the GC–MS technique [18, 19]. It results from the fact that the components of investigated sample, which are present in a mobile phase, leave the first chromatographic column and then they are collected and fractionally dosed into the second column using a modulator (being central element of the GCxGC system). Both columns differ in polarity of the stationary phase, which allows an increase in selectivity [20, 21]. The GCxGC–MS technique provides the information about qualitative and quantitative composition of the investigated samples characterized by complex matrix composition [22]. Waste generated in the wastewater treatment plants, including the biosolid cakes, belongs to aforementioned type of the samples, and hence, selection of the GCxGC technique is fully justified.

The aim of performed investigation was an attempt to identify those chemical compounds released from four types of biosolid cakes, which could be their potential markers. Identification of the compounds enabling differentiation between particular types of biosolids was based on the results of qualitative analysis employing the GCxGC–TOF–MS technique and analysis of variance. Quantitative analysis of the compounds and their olfactory thresholds provided theoretical odour concentrations for four investigated types of biosolid cakes.

## Results and discussion

### VOCs identified

Using the GCxGC–TOF–MS system, it was possible to identify the volatile chemical compounds emitted from various biosolid samples produced in WWTPs. As an example, Table 1 lists the chemical compounds emitted from the primary sludge. It can be observed that dominant groups of volatile substances emitted from the biosolid samples are: aldehydes, ketones, sulfur compounds, and aromatic hydrocarbons. These groups of VOCs have previously been reported in the other studies [23–25]. However, in some cases, the chemical compounds belonging to different classes can have similar physicochemical properties and it causes a co-elution in the first retention time. As a result, it is not possible to separate all of the substances present in analysed samples. Application of two-dimensional gas chromatography system allows obtaining full separation of determined compounds. It is a consequence of using two independent separation mechanisms based on volatility and polarity.

**Table 1:** Chemical compounds emitted from primary sludge

1st RT/s	2nd RT/s	Name of compounds	Similarity^a^
270	1.46	2-Propenal	876
278	1.22	Methanethiol	881
286	2.19	Acetonitrile	966
286	1.74	Ethanol	939
290	1.38	2-Propanone	926
290	1.46	2-Propenal	875
290	1.32	Propanal	894
294	1.21	Ethanethiol	907
302	1.33	Dimethyl sulfide	919
310	2.31	1-Propanol	937
318	1.33	Diethyl disulfide	918
326	1.66	2-Butanone	946
330	1.55	Butanal	952
350	1.54	2-Ethylbutanal	970
370	1.66	Benzene	901
374	1.28	2-Hexanone	906
378	1.18	Pentanal	876
386	1.55	3-Ethyl-3-pentanol	939
446	2.16	Dimethyl disulfide	973
478	1.89	Toluene	933
478	2.98	Pyridine	968
506	2.00	2-Methyl-3-heptanol	876
510	2.02	Hexanal	897
566	2.26	Methyl ethyl disulfide	879
578	1.22	Butane	863
614	2.00	Ethylbenzene	940
626	2.04	1,4-Dimethylbenzene	960
650	2.20	2-Heptanone	972
658	2.15	Methyl propyl disulfide	916
666	2.16	Heptanal	902
666	2.20	1,2-Dimethylbenzene	895
694	1.26	Nonane	931
706	1.31	1-Decanol	854
750	1.24	Decane	878
754	1.42	α-Pinene	927
762	2.02	Benzaldehyde	966
766	1.91	2-Ethylhexanal	931
778	1.51	Camphene	874
794	3.65	Benzonitrile	937
822	1.28	3-Ethyloctane	885
826	2.24	2-Octanone	940
846	2.20	Octanal	943
858	1.19	Phenol	874
874	1.64	α-Phellandrene	870
894	2.39	1,3,5-Trimethylbenzene	934
918	1.68	Limonene	929
954	1.90	Acetophenone	937
970	1.72	α-Terpinene	910
978	2.09	1-Methyl-2-propylbenzene	963
1050	2.82	3-Pentanol	865
1234	3.88	Benzothiazole	897
1514	1.46	Acetic acid, decyl ester	921

^a^Similarity: average of similarity of the mass spectrum of identified compounds to the spectra of standard compounds in the NIST 2011 library

Percentage contributions of chemical classes in the tested biosolid samples are presented in Table 2. It can be observed that sulfur compounds are dominant group of the chemical compounds in primary and dewatered sludge (28 and 25%, respectively). In waste-activated sludge, aliphatic hydrocarbons, alcohols, and ketones are the most significant groups of chemical compounds. Dominant group of the chemical compounds in digested sludge is aliphatic hydrocarbons. Changes in the percentage contribution of chemical classes in various types of biosolids are strongly related to operation of WWTPs. During biological treatment, percentage contribution of sulfur compounds decreases, but sludge dewatering operation results in a significant increase in concentration of these compounds. Percentage contribution of aromatic hydrocarbons, which are one of the dominant groups of chemical compounds in the biosolid samples, is very similar for each type of sludge (from 12% in waste-activated sludge to 19% in dewatered sludge). Similarly, percentage contribution of alcohols does not change significantly during sludge treatment (from 16% in dewatered sludge to 21% in waste-activated sludge).

**Table 2:** Percentage contributions of chemical classes in the tested biosolid samples

Chemical classes	Primary sludge (%)	Waste-activated sludge (%)	Digested sludge (%)	Dewatered sludge (%)
Sulfur compounds	28	9	7	25
Aromatic hydrocarbons	16	12	14	19
Aldehydes	10	9	11	6
Ketones	9	20	11	9
Aliphatic hydrocarbons	9	20	25	16
Alcohols	19	21	18	16
Others	9	9	14	9

### Estimation of key odoriferous compounds emitted from biosolid cakes

Table 3 presents surface area of the chromatographic peaks obtained for 20 chemical compounds, identified in volatile fraction of the investigated biosolid cakes, which have the biggest influence on the result of the performed statistical analysis. These compounds were divided into four chemical classes: alcohols, ketones, aromatic compounds, and organosulfur compounds, to find some dependences occurring within particular groups of the compounds. The statistical analysis revealed that each of the four types of biosolid cakes had different, statistically significant, composition of the volatile fraction. Significant differences among volatiles in each biosolid sample were indicated using small letter:

**Table 3:** Differentiation between main VOCs emitted from biosolid samples; mean values (*n* = 3) ± SD; different letters in the same line indicate significant differences among volatiles in each biosolid samples (Tukey’s HSD test. *P* < 0.05)

Chemical compound	Chromatographic peak area/mean ± SD (× 10^− 3^)			
	PS^a^	WAS^a^	DGS^a^	DWS^a^
Alcohols				
1-Decanol	20.5 ± 4.1	18.5 ± 3.8	35.4 ± 2.3	34.5 ± 1.6
2-Methyl-3-heptanol	12.8 ± 2.6^a,c^	46.1 ± 4.8^a,d,e^	11.5 ± 1.5^d,f^	74.5 ± 3.5^c,e,f^
Ethanol	47.4 ± 2.7^a^	851.1 ± 23.2^a^	11.0 ± 1.0^a^	231.8 ± 2.7^a^
1-Propanol	601.7 ± 1.8^a^	720.4 ± 0.8^a^	93.9 ± 5.6^a^	191.9 ± 3.4^a^
3-Ethyl-3-pentanol	14.2 ± 1.2	17.1 ± 2.0	16.3 ± 1.1	15.9 ± 0.8
Ketones				
2-Propanone	46.7 ± 2.3^c^	47.7 ± 3.3^e^	47.2 ± 1.2^f^	177.1 ± 6.0^c,e,f^
Acetophenone	263.1 ± 15.1^b,c^	278.9 ± 6.3^d,e^	64.9 ± 2.6^b,d,f^	19.8 ± 1.6^c,e,f^
2-Hexanone	787.3 ± 5.2^a^	830.9 ± 3.0^a^	55.3 ± 2.5^a^	86.8 ± 1.4^a^
2-Heptanone	20.5 ± 1.0^b^	12.6 ± 2.1^d^	72.3 ± 4.7^b.d.f^	22.4 ± 2.7^f^
2-Octanone	47.1 ± 1.7^a^	26.8 ± 2.2^a^	44.4 ± 2.3	30.6 ± 0.9
Aromatic compounds				
Benzene	884.6 ± 10.5^a,b^	930.7 ± 6.9^a,d,e^	906.0 ± 1.3^b,d^	890.9 ± 6.8^e^
1.3.5-Trimethylbenzene	21.3 ± 2.3^c^	6.9 ± 0.5^e^	14.7 ± 2.0^f^	89.8 ± 1.8^c,e,f^
*o*-Xylene	69.1 ± 0.8^b,c^	68.1 ± 1.0^d,e^	152.5 ± 6.0^b,d,f^	190.5 ± 8.4^c,e,f^
*p*-Xylene	51.2 ± 4.2^b^	45.8 ± 2.9^d^	172.8 ± 4.4^b,d,f^	37.0 ± 3.0^f^
Toluene	85.0 ± 1.4^b,c^	66.2 ± 2.5^d,e^	199.4 ± 2.6^b,d,f^	750.9 ± 1.8^c,e,f^
Sulfur compounds				
Ethanethiol	963.6 ± 7.0^a^	53.6 ± 0.2^a^	653.1 ± 5.5^a^	545.8 ± 1.3^a^
Dimethyl sulfide	43.9 ± 2.2^a^	14.9 ± 1.7^a^	222.9 ± 3.9^a^	650.8 ± 2.1^a^
Dimethyl disulfide	99.4 ± 3.9^a^	23.6 ± 2.1^a^	893.4 ± 5.5^a^	248.0 ± 0.4^a^
Diethyl disulfide	13.0 ± 1.2^b,c^	7.5 ± 0.9^d,e^	768.6 ± 1.8^b,d,f^	985.5 ± 4.7^c,e,f^
Methanethiol	2888.8 ± 4.8^a^	90.7 ± 1.2^a^	110.9 ± 1.9^a^	771.4 ± 1.8^a^

^a^Differences between PS and WAS

^b^Differences between PS and DGS

^c^Differences between PS and DWS

^d^Differences between WAS and DGS

^e^Differences between WAS and DWS

^f^Differences between DGS and DWS

- The letter “a” indicates significant differences between primary sludge and waste-activated sludge (PS–WAS);
- The letter “b” indicates significant differences between primary sludge and digested sludge (PS–DGS);
- The letter “c” indicates significant differences between primary sludge and dewatered sludge (PS–DWS);
- The letter “d” indicates significant differences between waste-activated sludge and digested sludge (WAS–DGS);
- The letter “e” indicates significant differences between waste-activated sludge and dewatered sludge (WAS–DWS);
- The letter “f” indicates significant differences between digested sludge and dewatered sludge (DGS–DWS).

The letter “a” located within the same line indicates significant differences between all four types of biosolid samples.

Based on the data presented in Table 3, it can be stated that, within the chemical class of alcohols, only two compounds (ethanol and 1-propanol) exhibited statistically significant differences in composition of volatile fraction of the investigated biosolid cakes. 1-Propanol was proposed as a potential marker, because the presence of ethanol can result from numerous processes and operations, conducted not only in the wastewater treatment plants. 1-Propanol is one of the most frequently identified alcohols released from the biosolid cakes [24]. 2-Hexanone can be proposed as a potential marker from the ketones’ group. Similar to 1-propanol, it can be the marker allowing differentiation between various biosolid cakes.

In case of the statistical analysis of aromatic hydrocarbons, it is difficult to point a single compound, which could be a potential marker for biosolid cake differentiation. Toluene is the compound, which makes it possible to discriminate almost all investigated biosolid cakes—an exception is differentiation between primary and excess biosolid cakes, in which content of this substance is similar. Analogous situation occurs for the isomers of *o*-xylene and *p*-xylene. However, differentiation between primary and excess biosolid cakes is possible with benzene. It is worth emphasizing that the papers concerning measurement of the compounds emitted from the wastewater treatment plants show that toluene as well as isomers of xylene are the compounds characteristic for biosolid cake processing [24, 26].

The last group included in Table 3 is organosulfur compounds. Many papers on investigation of the biosolid cakes found these substances as key ones [25, 27, 28]. Performed statistical analysis revealed that methanethiol and ethanethiol could be the potential markers of particular types of the biosolid cakes. This statement is supported by the fact that remaining three compounds from the organosulfur group allow discrimination of most biosolid cakes, except for primary and excess ones.

Table 4 gathers concentrations of the odoriferous compounds, which were proposed, based on the statistical analysis, as potential markers of four types of biosolid cakes. Analysis of these data indicates that organosulfur compounds are characterized by relatively low concentrations as compared to the remaining compounds released from the biosolid cakes, especially in case of the excess cake. It can be also observed that the biosolid cakes after fermentation process exhibit higher content of organosulfur compounds than the excess cake. Literature explains this phenomenon by the fact that protein amino acids (for instance methionine), responsible for the production of organosulfur compounds in anaerobic conditions, are relatively durable and they do not undergo degradation [27, 29].

**Table 4:** Concentration of key chemical compounds emitted from biosolid samples

Chemical compound	C ± SD/ppb				Odour description	OT/ppb [31]
	PS	WAS	DGS	DWS		
1-Propanol	23.0 ± 1.2	28.2 ± 1.3	3.9 ± 0.2	74.1 ± 2.9	Alcoholic, fruity, musty, pungent	94.0
2-Hexanone	19.5 ± 0.9	20.7 ± 1.1	1.4 ± 0.2	2.2 ± 0.1	Ethereal, fruity, cinnamon	24.0
*o*-Xylene	15.8 ± 0.7	15.6 ± 0.6	34.9 ± 1.7	43.7 ± 2.1	Fatty, geranium, oily, pungent	380.0
*p*-Xylene	11.7 ± 0.7	10.3 ± 0.5	39.5 ± 1.2	8.5 ± 0.4	Cold meat fat, sweet	58.0
Toluene	2.2 ± 0.1	1.7 ± 0.3	5.2 ± 0.3	19.5 ± 0.9	Ethereal, pungent, rubber, solvent,	330.0
Ethanethiol	3.7 ± 0.8	1.1 ± 0.1	2.5 ± 0.1	2.2 ± 0.1	Garlic-like, skunk-like, strong	0.0087
Dimethyl sulfide	1.6 ± 0.2	0.5 ± 0.1	8.5 ± 0.3	25.1 ± 1.5	Cabbage, mouldy, sulfurous	3.0
Dimethyl disulfide	2.5 ± 0.3	0.6 ± 0.1	22.7 ± 1.3	6.3 ± 0.7	Cabbage, onion, sulfurous, putrid	2.2
Diethyl disulfide	0.3 ± 0.1	1.5 ± 0.1	15.1 ± 1.3	19.4 ± 1.7	Poultry, cabbage, brussels sprout	2.0
Methanethiol	14.4 ± 1.0	0.5 ± 0.1	0.6 ± 0.1	3.9 ± 0.2	Cabbage, rotten egg, sulfurous	0.07

Seven out of ten chemical compounds emitted from the biosolid cakes (presented in Table 3) reveal higher concentration in dewatered cake than in fermented cake. It can be caused by a rapid growth of bacteria following cake dewatering, especially when this process is carried out in centrifuges (these devices are used in the treatment plants, from which the investigated samples of biosolid cakes were collected) [30].

Substantial differences were observed upon comparison of the results of VOCs emission from dewatered biosolid cakes originating from the treatment plants with different systems of water removal. One of them concerned aromatic hydrocarbons content in dewatered biosolid cakes—the cakes dewatered in a mechanical way using the centrifuges were richer in aromatic hydrocarbons than gravitationally dewatered cakes [24]. Another factors, which can have an influence on emission level of particular compounds from dewatered biosolid cakes, are the parameters of fermentation process (temperature and retention time) and, in the case of cake dewatering, amount of added flocculant, which facilitates separation of solid particles from liquid fraction via participation in aggregates’ formation [29].

**Fig. 1:**
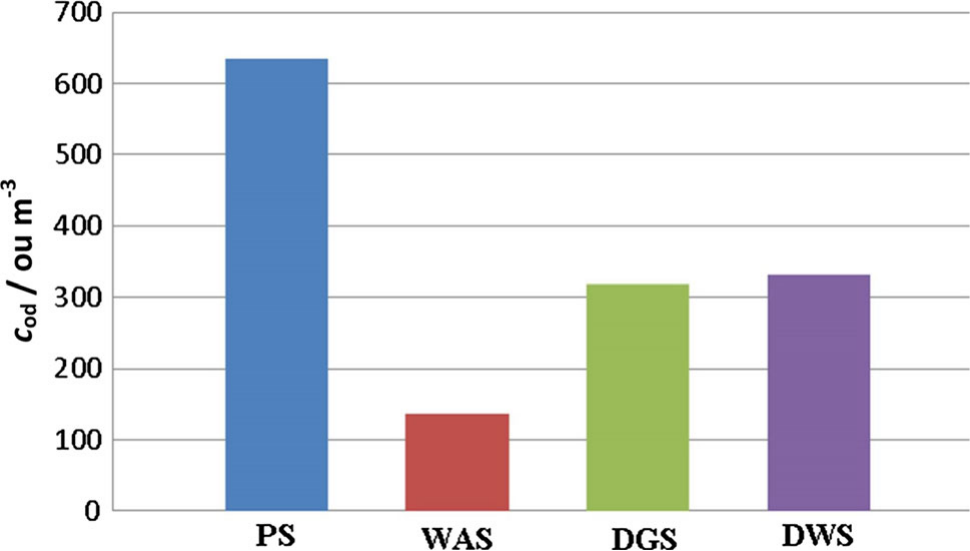
Odor concentration of biosolid samples

Table 4 also presents the values of olfactory threshold (OT), together with odour description. Olfactory threshold is the lowest concentration of each substance, which causes sensing of the smell with 50% probability. These data were used to determine theoretical odour concentrations for each of the four investigated types of biosolid cakes. The theoretical odour concentrations were calculated as a ratio of concentration of particular compound to its olfactory threshold. Figure 1 illustrates the results obtained in this way. The highest value of *c*_*od*_ was obtained for the primary cake, whereas the lowest one was for the excess cake. This situation is connected primarily with the concentration level of organosulfur compounds in these two types of biosolid cakes. Much higher content of those compounds is in the primary cake. Organosulfur compounds reveal very low values of olfactory threshold; hence, their contribution to strength of perceived odour is decisive. It is also the reason why dewatered cake exhibits higher odour concentration than fermented cake. One of the problems connected with the determination of concentration of organosulfur compounds emitted from the biosolid cakes is their instability. In the case of application of the solid sorbent-filled tubes, these compounds are released due to thermal desorption (at ca. 250–300 °C). At this temperature, thiols can be converted into sulfides. When thiols are present below LOD, it is reasonable to take advantage of summary concentration of organosulfur compounds not to omit contribution of such compounds as methanethiol or ethanethiol to strength of perceived odour [30, 32]. However, this approach hinders determination of the contribution of particular odorants because of different values of olfactory threshold.

## Conclusions

Application of the methodology proposed in this paper, based on the GCxGC–TOF–MS technique and statistical analysis, allowed the determination of concentration of the volatile organic compounds, which could be potential markers for differentiation between four main types of biosolid cakes. Employed procedure was much less time-consuming mainly due to no need for quantitative analysis of all identified compounds, which in the case of gas samples is usually long and laborious step. Two-dimensional gas chromatography coupled with time-of-flight mass spectrometry technique makes it possible to define chemical composition of the samples with complex matrix, including such biological samples as the biosolid cakes. Utilization of numerical values of olfactory thresholds allows assessment of perceived odour strength of the biosolid cake samples, without olfactometric analyses. A disadvantage of the olfactometric approach is a need for team of trained assessors and suitable laboratory. The methodology presented in the paper can be an alternative to the existing solutions in the field of measurement of VOCs’ emission level from the biosolid cakes.

## Experimental

### Biosolid sample collection

The biosolid samples were collected from the WWTP “Debogórze” located in Pomeranian Voivodeship, Poland. This facility is one of the biggest plants in this region and consists of three main technological sections: mechanical section, biological section, and sludge treatment section. Each day, 55,000 m^3^ of sewage are supplied to this facility, 1.5 tons of waste are produced during mechanical treatment, and 31 tons of solid sludge are generated.

Four biosolid samples (primary sludge—PS; waste-activated sludge—WAS; digested sludge—DGS; and dewatered sludge—DWS) were collected in 20 dm^3^ buckets and directly transported to a laboratory at the Gdansk University of Technology. Before measurements, the biosolid samples were stored at ambient conditions.

### Instrumentation

To generate emission from the biosolid samples, the US EPA flux hood chamber was used. This device is widely utilized for identification of the chemical compounds emitted from various types of surfaces. Before each measurement, the biosolid samples were purged for 30 min using nitrogen gas with the flow rate 5 dm^3^/min. The VOCs samples were collected for 10 min using the Tenax TA sorbent tubes (Gerstel, Germany), with the sampling flow rate 75 cm^3^/min.

The chemical compounds emitted from the biosolid samples were determined using two-dimensional gas chromatography (Agilent Technologies, Palo Alto, CA, USA) equipped with a liquid nitrogen-based dual-stage cryogenic modulator and coupled with Pegasus IV time-of-flight mass spectrometer (LECO Corp., St. Joseph, MI, USA). Equity 1 capillary column (30 m × 0.25 mm × 0.25 μm film, Supelco) was used as a primary column; SGWAX capillary column (2.0 m × 0.1 mm × 0.1 μm film, Agilent Technologies) was used as the second column. A modulation period of 5 s was employed. To separate all chemical components present in the samples, the following optimized temperature program was used: for the first GC oven: initial temperature of 40 °C maintained for 1 min, then ramped at 10 °C/min–90 °C and at 3 °C/min–240 °C, and, finally, kept constant for 5 min; for the secondary GC oven, the optimized temperature program was with the shift of + 5 °C regarding the program of the primary GC oven. Hydrogen at a constant flow rate 1 cm^3^/min was used as a carrier gas. The total analysis time was 45 min. Ions in the *m/z* = 40–500 range were analysed. The detector voltage was set to 1600 V and the temperature of the ion sources and the transfer line were maintained at 250 °C.

### Data analysis

Analysis of the data obtained with the GCxGC–TOF–MS system was performed using the algorithm for peak deconvolution (with signal-to-noise setup at 100), included in the Chroma TOF software (LECO Corp., USA, version 4.44) Tentative identification was accomplished based on the selected fragmentation ions listed in the NIST 2011 Mass Spectral Library v. 2.0).

### Statistical analysis

To determine the differences between volatile compounds emitted from various types of biosolids samples, two-way analysis of variance (ANOVA) was performed. Tukey’s honest significant difference test (HSD Tukey’s test, *p* < 0.05) was applied to compare the mean values of VOCs for different types of biosolid samples. The analysis was carried out using STATISTICA 12 software (StatSoft, Inc., Tulsa, Oklahoma, USA). Mean values and standard deviation (SD) were calculated from three repetitions for each biosolid sample.
